# Inflammatory Signatures of Graves’ Orbitopathy: Linking Thyroid Autoimmunity, Disease Activity, and Novel Hematological Biomarkers

**DOI:** 10.3390/diagnostics16132132

**Published:** 2026-07-07

**Authors:** Sadettin Ozturk, Elif Melis Baloğlu Akyol

**Affiliations:** Department of Endocrinology and Metabolism, Gaziantep City Hospital, Gaziantep 27100, Turkey; melis_baloglu@hotmail.com

**Keywords:** Graves’ disease, Graves’ orbitopathy, neutrophil-to-lymphocyte ratio, platelet-to-lymphocyte ratio, systemic immune-inflammation index, monocyte-to-HDL ratio, C-reactive protein-to-albumin ratio, thyroid autoimmunity, clinical activity score

## Abstract

**Background**: Graves’ disease is an autoimmune thyroid disorder that may be accompanied by systemic inflammation and Graves’ orbitopathy. This study evaluated the relationship between readily available hematological inflammatory markers and orbitopathy in patients with Graves’ disease. **Methods**: This retrospective observational study included 178 adult patients with Graves’ disease. Demographic, clinical, ophthalmological, and laboratory data were analyzed. Neutrophil-to-lymphocyte ratio (NLR), platelet-to-lymphocyte ratio (PLR), systemic immune-inflammation index (SII), monocyte-to-HDL cholesterol ratio (MHR), and C-reactive protein-to-albumin ratio (CAR) were calculated. Correlation, logistic regression, and ROC analyses were performed. **Results**: Among the 178 patients, 63 (35.4%) had Graves’ orbitopathy. Patients with orbitopathy had significantly higher NLR, PLR, SII, MHR, and CAR values than those without orbitopathy (all *p* < 0.001). Thyrotropin receptor antibody (TRAb) and thyroid-stimulating immunoglobulin (TSI) levels were positively correlated with all inflammatory markers. In multivariable logistic regression analysis, current smoking (OR 2.31, *p* = 0.047), TRAb (OR 1.08, *p* = 0.009), TSI (OR 1.06, *p* = 0.041), NLR (OR 1.63, *p* = 0.034), SII (OR 1.01, *p* = 0.018), MHR (OR 2.91, *p* = 0.012), and CAR (OR 3.84, *p* = 0.008) remained independently associated with Graves’ orbitopathy. Among the individual biomarkers, MHR showed the highest discriminative performance (AUC 0.818, 95% CI 0.754–0.882), while the combined inflammatory model achieved an AUC of 0.891 (95% CI 0.842–0.940), with an optimal predicted probability cut-off ≥ 0.43. **Conclusions**: Hematological inflammatory markers are associated with thyroid autoimmunity, disease activity, and Graves’ orbitopathy. These inexpensive and easily accessible markers may support clinical risk assessment in patients with Graves’ disease.

## 1. Introduction

Graves’ disease (GD) is the most common cause of hyperthyroidism and represents a complex autoimmune disorder characterized by the production of stimulatory autoantibodies directed against the thyroid-stimulating hormone receptor (TSHR). These antibodies induce excessive thyroid hormone synthesis and secretion, resulting in the clinical manifestations of thyrotoxicosis, diffuse goiter, and a wide spectrum of systemic symptoms [1,2]. Beyond thyroid dysfunction, Graves’ disease is increasingly recognized as a systemic immune-mediated disorder involving both innate and adaptive immune responses, which contribute to persistent inflammation and tissue-specific manifestations such as Graves’ orbitopathy and dermopathy [3,4].

The pathogenesis of Graves’ disease involves a multifaceted interaction between genetic susceptibility, environmental triggers, and immune dysregulation. Activation of autoreactive T lymphocytes stimulates B-cell differentiation and autoantibody production, leading to sustained immune activation [5,6]. Pro-inflammatory cytokines, including tumor necrosis factor-alpha (TNF-α), interferon-gamma (IFN-γ), interleukin-6 (IL-6), and interleukin-17 (IL-17), play central roles in disease initiation and progression [7,8,9,10]. These cytokines not only promote thyroid autoimmunity but also influence peripheral blood cell populations, resulting in measurable alterations in neutrophil, lymphocyte, and platelet counts [11].

Among these indices, the neutrophil-to-lymphocyte ratio (NLR), platelet-to-lymphocyte ratio (PLR), and systemic immune-inflammation index (SII) have gained considerable attention as inexpensive and readily available biomarkers reflecting the balance between innate and adaptive immunity [12,13]. Neutrophils participate actively in inflammatory responses through cytokine secretion, oxidative burst activity, and tissue infiltration, whereas lymphocyte counts may decrease during periods of heightened systemic stress and immune activation. Platelets additionally contribute to inflammation by releasing chemokines and facilitating leukocyte recruitment [14,15].

Over the past decade, numerous studies have demonstrated the prognostic and diagnostic significance of hematological inflammatory markers in autoimmune, cardiovascular, oncological, and endocrine disorders [16,17]. In thyroid diseases, particularly autoimmune thyroid disorders, increasing evidence suggests that these indices may reflect disease activity, severity of immune response, and treatment outcomes. Previous investigations have reported significantly elevated NLR and PLR levels in patients with Graves’ disease compared with healthy controls, suggesting the presence of systemic inflammatory activation beyond thyroid hormone excess alone [18,19,20]. Furthermore, some studies have observed correlations between hematological inflammatory markers and thyroid hormone concentrations, thyroid autoantibody titers, and clinical manifestations of the disease [21,22].

Graves’ orbitopathy is the most common extrathyroidal manifestation of Graves’ disease and reflects persistent immune activation and systemic inflammation. However, the mechanisms underlying the development of orbitopathy in only a subset of patients remain incompletely understood. Therefore, systemic inflammatory markers may provide useful insights into disease activity and the underlying inflammatory processes [23].

Routine laboratory assessment of Graves’ disease primarily focuses on thyroid function tests and thyroid-specific autoantibodies, including thyroid-stimulating hormone (TSH), free triiodothyronine (FT3), free thyroxine (FT4), and TSH receptor antibodies (TRAb). However, these biomarkers may not fully reflect the systemic inflammatory component of the disease. Hematological inflammatory indices are inexpensive, widely available, and readily calculated from routine laboratory tests, making them attractive complementary biomarkers for assessing immune activation and inflammatory burden [24,25].

Despite growing interest in hematological inflammatory markers, evidence regarding their association with thyroid autoimmunity, disease activity, and Graves’ orbitopathy remains limited. Although previous studies have demonstrated elevated inflammatory indices in Graves’ disease, their relationships with thyroid autoantibodies and clinical manifestations have not been fully clarified. A better understanding of these associations may improve disease assessment and facilitate the identification of practical biomarkers for routine clinical use.

Therefore, this study aimed to evaluate hematological inflammatory markers, including NLR, PLR, SII, MHR, and CAR, in patients with Graves’ disease and to investigate their associations with thyroid function, thyroid autoantibodies, disease activity, and Graves’ orbitopathy. By comparing patients with and without orbitopathy, we sought to determine the potential clinical utility of these readily available inflammatory indices as complementary biomarkers for disease assessment and risk stratification.

## 2. Materials and Methods

### 2.1. Study Design and Population

The study protocol was reviewed and approved by the Gaziantep City Hospital Non-Interventional Clinical Research Ethics Committee (Approval No: 420/2026; Approval Date: 21 January 2026). All procedures were performed in accordance with the ethical principles of the Declaration of Helsinki and its subsequent amendments.

This retrospective observational study was conducted at the Department of Endocrinology and Metabolic Diseases, Gaziantep City Hospital, Türkiye. The study evaluated hematological inflammatory parameters and their association with disease activity and clinical findings in patients diagnosed with Graves’ disease.

Patients aged 18 years or older who were diagnosed with Graves’ disease and followed in the outpatient endocrinology clinic between January 2020 and December 2025 were screened for eligibility. The diagnosis of Graves’ disease was established based on the presence of biochemical hyperthyroidism, suppressed serum TSH levels, elevated FT4 and/or FT3 concentrations, and positive TRAb levels, in accordance with the 2016 American Thyroid Association (ATA) Guidelines for Diagnosis and Management of Hyperthyroidism and Other Causes of Thyrotoxicosis [24,26].

Patients were eligible for inclusion if they were aged 18 years or older, had a confirmed diagnosis of Graves’ disease, and had available thyroid function tests and complete blood count measurements at the time of diagnosis. In addition, only patients with complete clinical records regarding disease characteristics, treatment modalities, and follow-up assessments were included in the study. Patients were excluded if they had evidence of active infection at the time of laboratory evaluation, a history of hematological disorders affecting blood cell counts, malignancy, other systemic autoimmune or inflammatory diseases, chronic immunosuppressive therapy unrelated to Graves’ disease, or incomplete clinical or laboratory data.

### 2.2. Data Collection and Variables

Demographic, clinical, and laboratory data were retrospectively obtained from the hospital electronic medical records system. Demographic characteristics included age, sex, and smoking status. Clinical variables comprised the date of Graves’ disease diagnosis, duration of follow-up, treatment modality (antithyroid drugs, radioactive iodine therapy, or thyroidectomy), and the presence of Graves’ orbitopathy. Graves’ orbitopathy was diagnosed according to the recommendations of the European Group on Graves’ Orbitopathy (EUGOGO) based on clinical findings and ophthalmological evaluations documented in the medical records [27]. Laboratory data collected at the time of diagnosis included TSH, FT3, FT4, and TRAb levels, and complete blood count parameters, including neutrophil, lymphocyte, and platelet counts.

Hematological inflammatory indices were calculated using complete blood count parameters. The neutrophil-to-lymphocyte ratio (NLR) was calculated by dividing the absolute neutrophil count by the absolute lymphocyte count, the platelet-to-lymphocyte ratio (PLR) by dividing the platelet count by the lymphocyte count, and the systemic immune-inflammation index (SII) using the formula: platelet count × neutrophil count/lymphocyte count. The monocyte-to-HDL cholesterol ratio (MHR) was calculated by dividing the monocyte count by HDL cholesterol level. The C-reactive protein-to-albumin ratio (CAR) was calculated by dividing CRP concentration by serum albumin level. These indices were calculated using laboratory measurements obtained at the time of diagnosis. All laboratory measurements and hematological inflammatory markers included in the primary analyses were obtained from blood samples collected at the time of diagnosis, before initiation of antithyroid treatment. No follow-up laboratory measurements were included in the correlation, logistic regression, or ROC analyses.

### 2.3. Outcome Measures

The primary outcome was to evaluate the association of hematological inflammatory markers with Graves’ orbitopathy and disease activity. Secondary outcomes included their relationships with thyroid function parameters and their diagnostic performance for identifying orbitopathy.

### 2.4. Statistical Analysis

All statistical analyses were performed using IBM SPSS Statistics version 27.0 (IBM Corp., Armonk, NY, USA) and R software (version 4.3.0; R Foundation for Statistical Computing, Vienna, Austria). Continuous variables were assessed for normality using the Kolmogorov–Smirnov test and visual inspection of histograms and Q-Q plots. Normally distributed variables were expressed as mean ± standard deviation (SD), whereas non-normally distributed variables were presented as median (interquartile range [IQR]). Categorical variables were expressed as frequencies and percentages. Comparisons of baseline measurements were performed using the paired-samples *t*-test for normally distributed variables and the Wilcoxon signed-rank test for non-normally distributed variables. Comparisons between patients with and without Graves’ orbitopathy were performed using the independent samples *t*-test or Mann–Whitney U test for continuous variables and the chi-square test or Fisher’s exact test for categorical variables, as appropriate. Correlations between hematological inflammatory parameters (NLR, PLR, and SII) and thyroid-related biochemical markers (TSH, FT3, FT4, and TRAb) were evaluated using Spearman’s rank correlation analysis. To identify independent predictors of Graves’ orbitopathy, variables demonstrating a *p*-value < 0.10 in univariable analyses were entered into a multivariable logistic regression model. Odds ratios (ORs) and 95% confidence intervals (CIs) were reported. Variance inflation factors (VIFs) were calculated prior to multivariable modeling and no significant multicollinearity was observed (VIF < 5). A two-sided *p*-value < 0.05 was considered statistically significant.

## 3. Results

**Figure 1:**
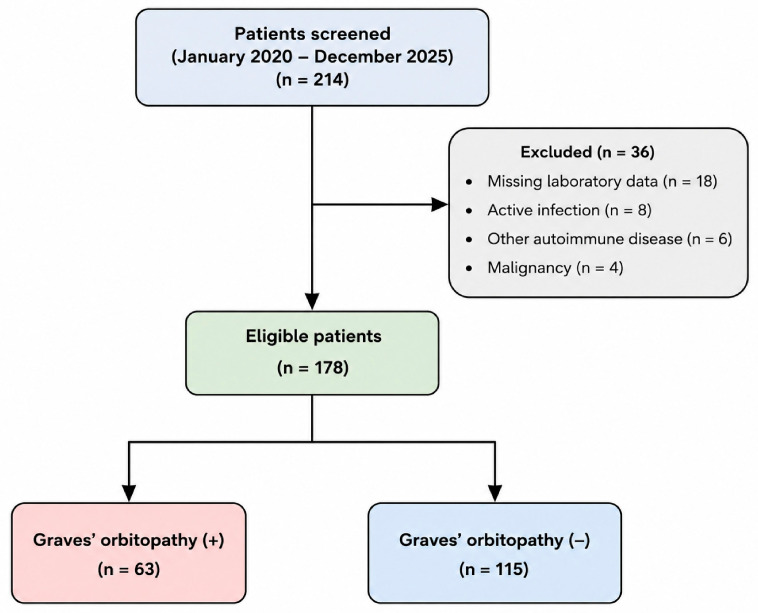
Flow diagram illustrating patient selection and allocation into Graves’ orbitopathy (+) and Graves’ orbitopathy (−) groups.

A total of 214 patients diagnosed with Graves’ disease between January 2020 and December 2025 were initially identified from the endocrinology database. After applying the exclusion criteria, 36 patients were excluded due to missing laboratory data (*n* = 18), active infection (*n* = 8), concomitant autoimmune diseases (*n* = 6), or malignancy (*n* = 4). Consequently, 178 eligible patients were included in the final analysis. Among these patients, 63 (35.4%) were diagnosed with Graves’ orbitopathy, whereas 115 (64.6%) did not have orbitopathy. The patient selection process is illustrated in Figure 1.

Baseline demographic, clinical, and thyroid-related characteristics of the study population are shown in Table 1. A total of 178 patients with Graves’ disease were included in the study, with a mean age of 41.35 ± 12.06 years. Females constituted the majority of the cohort (82.0%). Current smoking was reported in 18.5% of patients, while comorbidities were present in 8.4%, and TRAb positivity was observed in 86.5% of patients. Antithyroid drug therapy was administered to 93.8% of the cohort, predominantly methimazole (89.9%), whereas propylthiouracil, radioactive iodine therapy, and thyroidectomy were less frequently used. Graves’ orbitopathy was present in 63 patients (35.4%). The mean Clinical Activity Score (CAS) was 2.35 ± 1.71, while the mean right and left Hertel measurements were 21.02 ± 2.18 mm and 21.08 ± 2.09 mm, respectively. Additional clinical characteristics not shown in Table 1 included electronic cigarette use in 2 patients (1.1%), recurrent disease in 3 (1.7%), family history of thyroid disease in 19 (10.7%), statin use in 3 (1.7%), selenium treatment in 45 (25.3%), and steroid treatment in 11 (6.2%). The most frequent ophthalmological manifestations were spontaneous retrobulbar pain (19.7%), pain on up/down gaze (15.2%), conjunctival redness (13.5%), eyelid redness (10.7%), eyelid swelling (10.7%), caruncle/plica inflammation (7.9%), conjunctival edema (7.3%), and both diplopia and ocular motility restriction (11.2%) (Table 1).

The median thyroid volume was 11.38 mL (7.60–17.61), and the mean isthmus thickness was 4.86 ± 1.73 mm. Median TRAb and TSI levels at admission were 4.32 IU/L (2.33–9.87) and 4.00 (1.93–10.50), respectively, while median anti-TPO and anti-Tg levels were 226.05 IU/mL (38.47–681.12) and 6.05 IU/mL (0.93–40.98). Baseline thyroid function tests demonstrated median TSH, FT3, and FT4 values of 0.28 mIU/L (0.01–1.84), 3.71 pg/mL (3.25–4.94), and 0.82 ng/dL (0.67–1.42), respectively, with a median FT3/FT4 ratio of 3.99 (3.04–5.01). The mean HDL cholesterol level was 52.55 ± 13.83 mg/dL. Median neutrophil, lymphocyte, platelet, and monocyte counts were 4.17 × 10^9^/L (3.42–5.39), 2.59 ± 0.87 × 10^9^/L, 295.35 ± 83.52 × 10^9^/L, and 0.59 × 10^9^/L (0.48–0.70), respectively. Regarding inflammatory and biochemical indices, the mean albumin level was 42.54 ± 4.14 g/L, while the median CRP concentration was 2.50 mg/L (1.30–4.47). The median NLR was 1.78 (1.33–2.33), the mean PLR was 121.80 ± 43.98, the median SII was 495.94 (360.44–707.85), the mean MHR was 0.66 ± 0.60, and the median CAR was 0.06 (0.04–0.10) (Table 2).

Comparison of demographic, thyroid-related, ophthalmological, and inflammatory characteristics according to Graves’ orbitopathy status is shown in Table 3. Patients with Graves’ orbitopathy were older than those without orbitopathy (43.8 ± 11.7 vs. 40.0 ± 12.1 years, *p* = 0.038) and had a higher prevalence of current smoking (28.6% vs. 13.0%, *p* = 0.012). Disease duration was longer in the orbitopathy group (28 [12–54] vs. 16 [8–36] months, *p* = 0.011). TRAb and TSI levels were significantly higher among patients with orbitopathy, with median values of 8.92 IU/L (4.65–16.33) and 9.40 (4.10–18.60), respectively, compared with 3.14 IU/L (1.86–6.72) and 2.90 (1.40–7.50) in patients without orbitopathy (both *p* < 0.001). FT3 and FT4 concentrations were also higher in the orbitopathy group (*p* = 0.002 and *p* = 0.004, respectively). Clinical Activity Score was 3.82 ± 1.34 in patients with orbitopathy and 0.00 ± 0.00 in those without orbitopathy (*p* < 0.001). Right and left Hertel measurements were significantly greater in the orbitopathy group (both *p* < 0.001), while diplopia and ocular motility restriction were observed in 31.7% of patients with orbitopathy and were absent in patients without orbitopathy (both *p* < 0.001) (Table 3).

Regarding hematological parameters, patients with orbitopathy had higher neutrophil counts (4.91 [3.92–6.23] vs. 3.88 [3.21–4.87] × 10^9^/L, *p* < 0.001) and monocyte counts (0.66 [0.53–0.78] vs. 0.55 [0.45–0.67] × 10^9^/L, *p* = 0.001), whereas lymphocyte counts (2.31 ± 0.79 vs. 2.74 ± 0.88 × 10^9^/L, *p* = 0.003) and HDL cholesterol levels (49.1 ± 12.5 vs. 54.4 ± 14.2 mg/dL, *p* = 0.019) were lower. Inflammatory indices were significantly elevated in patients with orbitopathy, including NLR (2.18 [1.71–2.98] vs. 1.53 [1.20–1.97]), PLR (139.6 ± 46.5 vs. 112.8 ± 39.2), SII (692.4 [498.2–955.6] vs. 418.6 [311.5–578.2]), MHR (0.89 ± 0.48 vs. 0.54 ± 0.32), and CAR (0.10 [0.06–0.17] vs. 0.05 [0.03–0.08]), with all comparisons yielding *p* < 0.001 (Table 3).

Spearman correlation analysis demonstrated significant positive correlations between all inflammatory markers and thyroid autoimmunity parameters TRAb and TSI. The strongest correlations with TRAb were observed for SII (r = 0.431, *p* < 0.001), MHR (r = 0.417, *p* < 0.001), NLR (r = 0.382, *p* < 0.001), and CAR (r = 0.351, *p* < 0.001). Similarly, TSI showed significant positive correlations with SII (r = 0.402, *p* < 0.001), MHR (r = 0.388, *p* < 0.001), NLR (r = 0.346, *p* < 0.001), PLR (r = 0.271, *p* < 0.001), and CAR (r = 0.322, *p* < 0.001). In contrast, TSH demonstrated weak but significant negative correlations with NLR (r = −0.219, *p* = 0.004), PLR (r = −0.171, *p* = 0.023), SII (r = −0.248, *p* = 0.001), MHR (r = −0.204, *p* = 0.007), and CAR (r = −0.183, *p* = 0.015) (Table 4).

FT3 and FT4 levels were positively correlated with all inflammatory markers, with the strongest associations observed between FT3 and SII (r = 0.335, *p* < 0.001) and between FT4 and SII (r = 0.301, *p* < 0.001). No significant correlations were identified between inflammatory markers and anti-TPO, anti-Tg, or the FT3/FT4 ratio. Clinical Activity Score (CAS) exhibited moderate positive correlations with NLR (r = 0.446, *p* < 0.001), PLR (r = 0.341, *p* < 0.001), SII (r = 0.523, *p* < 0.001), MHR (r = 0.478, *p* < 0.001), and CAR (r = 0.412, *p* < 0.001). Likewise, right and left Hertel measurements showed significant positive correlations with all inflammatory indices, with the strongest relationships observed for MHR (right Hertel: r = 0.352, *p* < 0.001; left Hertel: r = 0.361, *p* < 0.001) and SII (right Hertel: r = 0.387, *p* < 0.001; left Hertel: r = 0.394, *p* < 0.001) (Table 4).

**Figure 2:**
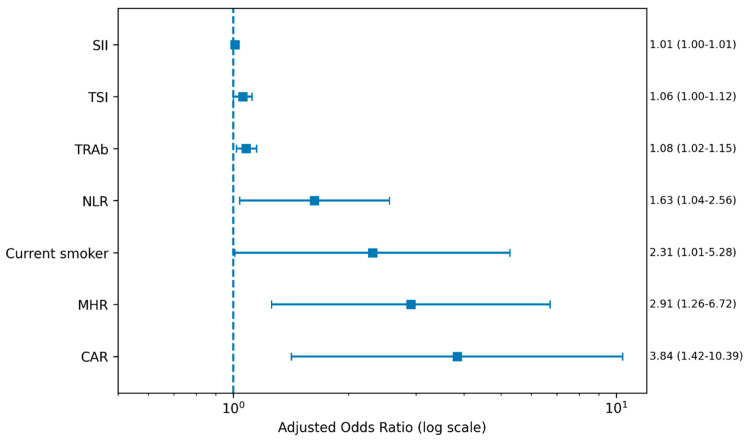
Forest plot of multivariable logistic regression analysis identifying independent predictors of Graves’ orbitopathy.

In the univariable logistic regression analysis, age, current smoking, TRAb, TSI, FT3, FT4, NLR, PLR, SII, MHR, and CAR were significantly associated with Graves’ orbitopathy (Table 5). In the multivariable logistic regression analysis, current smoking, TRAb, TSI, NLR, SII, MHR, and CAR remained independent predictors of Graves’ orbitopathy, whereas age, FT3, FT4, and PLR were no longer statistically significant after adjustment for the other covariates (Table 5). These findings are visually summarized in Figure 2.

**Figure 3:**
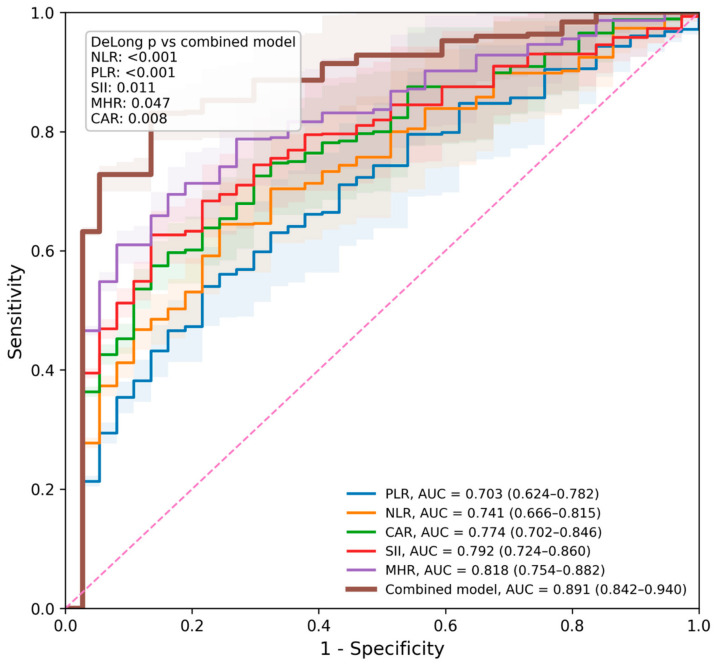
ROC curves of NLR, PLR, SII, MHR, and CAR for identifying Graves’ orbitopathy.

ROC analysis demonstrated that all inflammatory markers significantly predicted Graves’ orbitopathy (all *p* < 0.001), as illustrated in Figure 3. Among the individual markers, MHR showed the highest discriminative performance with an AUC of 0.818 (95% CI: 0.754–0.882), followed by SII with an AUC of 0.792 (95% CI: 0.724–0.860), CAR with an AUC of 0.774 (95% CI: 0.702–0.846), NLR with an AUC of 0.741 (95% CI: 0.666–0.815), and PLR with an AUC of 0.703 (95% CI: 0.624–0.782). The optimal cut-off values were 1.89 for NLR, 126.5 for PLR, 562.4 for SII, 0.74 for MHR, and 0.08 for CAR. Sensitivity and specificity values ranged from 68.3% to 79.4% and from 63.5% to 73.0%, respectively. The combined inflammatory model, incorporating NLR, SII, MHR, and CAR, achieved an AUC of 0.891 (95% CI: 0.842–0.940), with a sensitivity of 84.1%, specificity of 82.6%, and a Youden index of 0.667. The optimal predicted probability cut-off for the combined model was ≥0.43.

DeLong comparison analyses showed that the combined model significantly outperformed each individual inflammatory marker. The differences in AUC between the combined model and NLR, PLR, SII, MHR, and CAR were 0.150 (*p* < 0.001), 0.188 (*p* < 0.001), 0.099 (*p* = 0.011), 0.073 (*p* = 0.047), and 0.117 (*p* = 0.008), respectively (Table 6, Figure 3).

**Figure 4:**
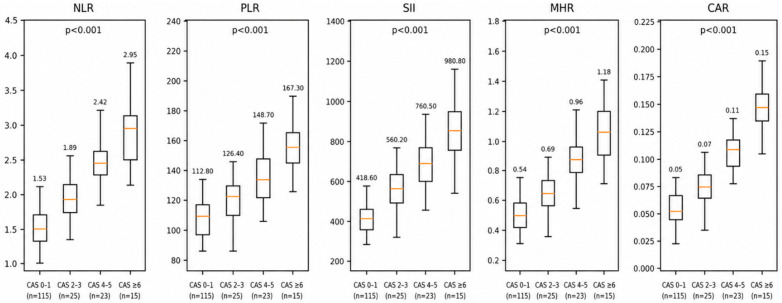
Distribution of NLR, PLR, SII, MHR, and CAR according to CAS categories.

The distribution of inflammatory markers according to Clinical Activity Score (CAS) categories is shown in Table 7. Progressive increases were observed across higher CAS categories for all evaluated inflammatory indices. Median NLR values increased from 1.53 (1.20–1.97) in patients with CAS 0–1 to 1.89 (1.45–2.41) in those with CAS 2–3, 2.42 (1.90–3.15) in those with CAS 4–5, and 2.95 (2.20–3.80) in patients with CAS ≥ 6 (*p* < 0.001). Similarly, mean PLR values increased from 112.8 ± 39.2 to 126.4 ± 41.7, 148.7 ± 44.5, and 167.3 ± 48.2 across the respective CAS categories (*p* < 0.001). Median SII values were 418.6 (311.5–578.2), 560.2 (430–710), 760.5 (590–980), and 980.8 (760–1250), respectively (*p* < 0.001). Mean MHR values increased from 0.54 ± 0.32 in the lowest CAS category to 0.69 ± 0.37, 0.96 ± 0.49, and 1.18 ± 0.55 in progressively higher CAS categories (*p* < 0.001). Likewise, median CAR values increased from 0.05 (0.03–0.08) to 0.07 (0.04–0.11), 0.11 (0.07–0.18), and 0.15 (0.10–0.24) across increasing CAS categories (*p* < 0.001) (Table 7, Figure 4).

## 4. Discussion

The present study evaluated the relationship between hematological inflammatory markers and disease activity, thyroid autoimmunity, and Graves’ orbitopathy in a cohort of patients with Graves’ disease. The principal findings of this study were as follows: (i) patients with Graves’ orbitopathy exhibited significantly higher NLR, PLR, SII, MHR, and CAR values than those without orbitopathy; (ii) inflammatory markers demonstrated significant positive correlations with TRAb, TSI, Clinical Activity Score (CAS), and Hertel exophthalmometry measurements; (iii) NLR, SII, MHR, and CAR remained independent predictors of Graves’ orbitopathy after multivariable adjustment; (iv) increasing CAS categories were associated with progressively elevated inflammatory marker levels; and (v) a combined inflammatory model integrating NLR, SII, MHR, and CAR showed excellent discriminative performance for identifying Graves’ orbitopathy.

Graves’ disease is characterized by chronic immune activation involving both cellular and humoral immune pathways. Activated T lymphocytes, autoreactive B cells, macrophages, and pro-inflammatory cytokines contribute not only to thyroid dysfunction but also to extrathyroidal manifestations, particularly Graves’ orbitopathy [28,29,30]. Consequently, systemic inflammatory markers derived from routine hematological parameters may reflect the underlying immunological activity of the disease. In the present study, patients with orbitopathy demonstrated significantly elevated NLR, PLR, SII, MHR, and CAR values, supporting the concept that Graves’ orbitopathy represents a more pronounced inflammatory phenotype of Graves’ disease.

Previous studies have similarly reported associations between hematological inflammatory indices and autoimmune thyroid disorders. Atli et al. showed increased inflammatory hematological markers in Graves’ disease and proposed that these parameters may provide clinically useful information regarding disease activity [12]. Likewise, Turan et al. reported that patients with Graves’ disease exhibited significantly higher NLR values compared with healthy controls, suggesting that neutrophil predominance and relative lymphocyte suppression may reflect systemic inflammatory activation [31]. Our findings extend these observations by demonstrating that multiple inflammatory indices, including NLR, PLR, SII, MHR, and CAR, are not only elevated in Graves’ disease but are also closely associated with orbitopathy severity and activity.

One of the most important findings of the present study was the significant relationship between inflammatory markers and thyroid autoimmunity parameters. TRAb and TSI demonstrated consistent positive correlations with all evaluated inflammatory indices, particularly SII and MHR. These findings are biologically plausible because TRAb-mediated stimulation of orbital fibroblasts and thyroid tissue induces cytokine release, immune-cell recruitment, and chronic inflammatory responses [29,32,33]. Ezra et al. and Sun et al. reported the central role of IL-6- and IL-17-mediated inflammatory pathways in orbital tissue remodeling [32,33]. Philipp et al. [10] showed that macrophage infiltration and antigen-specific T-cell activation occur early during the development of experimental Graves’ orbitopathy [29]. The positive correlations observed between TRAb, TSI, and inflammatory markers in our study support the concept that systemic inflammatory activity parallels autoimmune activation in Graves’ disease.

Our results further demonstrated significant associations between inflammatory markers and thyroid hormone concentrations. FT3 and FT4 levels were positively correlated with NLR, PLR, SII, MHR, and CAR, whereas TSH exhibited negative correlations with these parameters. Haghbin et al. found significant relationships between inflammatory biomarkers and thyroid hormone status in autoimmune thyroid disease [34]. Hyperthyroidism is known to influence immune-cell dynamics, oxidative stress, and cytokine production, potentially contributing to elevations in hematological inflammatory indices. Therefore, the observed correlations likely reflect the combined effects of thyroid hormone excess and autoimmune inflammatory activation.

The relationship between inflammatory markers and clinical manifestations of Graves’ orbitopathy was particularly noteworthy. Clinical Activity Score demonstrated moderate positive correlations with all evaluated inflammatory indices, with the strongest correlation observed for SII. Furthermore, Hertel exophthalmometry measurements were significantly associated with NLR, PLR, SII, MHR, and CAR. These findings suggest that systemic inflammatory burden may parallel the severity of orbital involvement. Szydełko et al. showed associations between hematological inflammatory ratios and clinical characteristics of Graves’ orbitopathy, emphasizing the potential value of these markers in identifying patients with more active disease [35]. The progressive increase in inflammatory markers across CAS categories observed in our study further supports this relationship and suggests that these parameters may provide objective information regarding orbitopathy activity.

Multivariable logistic regression analysis demonstrated that current smoking, TRAb, TSI, NLR, SII, MHR, and CAR were independently associated with Graves’ orbitopathy. Smoking is a well-established risk factor for Graves’ orbitopathy and has consistently been linked to disease development, progression, and treatment resistance [27]. The independent associations observed for TRAb and TSI are also consistent with previous evidence identifying thyroid autoantibodies as key drivers of orbital inflammation [28,29]. Importantly, inflammatory indices remained significant even after adjustment for these established risk factors, suggesting that they capture additional information regarding disease-related inflammatory burden.

Among the evaluated biomarkers, CAR demonstrated the highest adjusted odds ratio in multivariable analysis. CAR integrates two complementary inflammatory parameters, CRP and albumin, and has emerged as a robust marker of systemic inflammation in various cardiovascular, oncological, and autoimmune disorders [17,36]. Additionally, MHR showed strong independent predictive performance. Monocytes are major producers of inflammatory cytokines and contribute to tissue remodeling, whereas HDL cholesterol exerts anti-inflammatory and antioxidant effects. Therefore, elevated MHR may reflect an imbalance between pro-inflammatory and protective mechanisms. The strong performance of CAR and MHR in the present study suggests that composite inflammatory indices may provide more comprehensive information than traditional hematological ratios alone.

ROC analyses further supported the clinical utility of inflammatory markers. Among individual biomarkers, MHR demonstrated the highest AUC, followed by SII and CAR. More importantly, the combined inflammatory model incorporating NLR, SII, MHR, and CAR achieved an AUC of 0.891, with significantly better discriminative performance than any individual marker. These findings indicate that different inflammatory pathways contribute complementary information regarding disease activity and orbitopathy risk. Similar observations have been reported in other inflammatory and autoimmune disorders, where multimarker approaches generally outperform single biomarkers [17,36]. The excellent performance of the combined model suggests that integrated inflammatory assessment may improve risk stratification in patients with Graves’ disease.

The biological mechanisms underlying these findings are likely multifactorial. Neutrophils contribute to inflammatory amplification through cytokine release and oxidative burst activity, while lymphocyte depletion may reflect chronic immune stress [14,37]. Platelets facilitate leukocyte recruitment and inflammatory signaling [15]. Monocytes participate directly in cytokine production and tissue remodeling within orbital tissues [29]. Simultaneously, elevated CRP and reduced albumin levels reflect systemic inflammatory activation. Consequently, composite indices such as SII, MHR, and CAR may capture multiple dimensions of immune dysregulation and provide a more comprehensive representation of disease activity.

The findings of this study suggest that readily available hematological inflammatory markers, including NLR, SII, MHR, and CAR, may provide valuable adjunctive information for the assessment of disease activity and the identification of patients at increased risk of Graves’ orbitopathy. Given their low cost, wide availability, and ease of calculation from routine laboratory tests, these biomarkers could complement conventional thyroid-specific parameters such as TRAb and TSI in clinical practice. Furthermore, the strong performance of the combined inflammatory model indicates that integrated inflammatory assessment may enhance risk stratification and support closer monitoring of patients with more active or severe disease.

Whether elevated inflammatory markers represent a cause or consequence of Graves’ orbitopathy remains uncertain. Given the retrospective cross-sectional nature of the present study, causality cannot be inferred. The observed elevations in NLR, SII, MHR, and CAR may reflect systemic immune activation secondary to Graves’ disease and orbital inflammation rather than directly contributing to disease development. Although these markers were higher in patients with established orbitopathy, the present dataset cannot determine whether they are already elevated in newly diagnosed patients without orbitopathy or whether high baseline values predict future orbitopathy. Therefore, these biomarkers should currently be interpreted as adjunctive markers of inflammatory burden and disease activity rather than definitive causal or predictive markers. Prospective longitudinal studies in newly diagnosed Graves’ disease patients without initial orbital involvement are needed to clarify whether these indices can be used for early risk stratification.

Although the causal and predictive roles of these biomarkers remain to be established, they may still have important clinical value in routine practice. From a practical perspective, the inflammatory markers evaluated in this study have several advantages over more specialized biomarkers. NLR, PLR, SII, MHR, and CAR are derived from routine complete blood count, lipid profile, CRP, and albumin measurements that are already obtained during the initial evaluation of most patients with Graves’ disease. Therefore, their calculation does not require additional laboratory testing or increase healthcare costs. Given their low cost, wide availability, and ease of calculation, these indices may represent cost-effective adjunctive tools for identifying patients at increased risk of Graves’ orbitopathy and for supporting clinical decision-making, particularly in resource-limited settings. Nevertheless, they should complement rather than replace established clinical, biochemical, and ophthalmological assessments until their predictive value is confirmed in prospective longitudinal studies.

Some limitations should be acknowledged. First, the retrospective single-center design may have introduced selection bias and limits causal inference. Second, inflammatory cytokines such as IL-6, TNF-α, and IL-17 were not available and therefore could not be evaluated alongside hematological markers. Third, external validation of the predictive models was not performed. Fourth, the relatively limited number of orbitopathy events may have affected model stability despite careful variable selection. Finally, longitudinal analyses evaluating dynamic changes in inflammatory markers following treatment were beyond the scope of the present investigation and should be addressed in future prospective studies. Despite these limitations, the study possesses several important strengths. It represents one of the few investigations simultaneously evaluating NLR, PLR, SII, MHR, and CAR in relation to thyroid autoimmunity, orbitopathy severity, and clinical activity. Furthermore, the inclusion of both correlation analyses and predictive modeling provides a comprehensive assessment of the clinical relevance of inflammatory biomarkers in Graves’ disease.

## 5. Conclusions

In conclusion, hematological inflammatory markers were significantly associated with thyroid autoimmunity, disease activity, and Graves’ orbitopathy in patients with Graves’ disease. Elevated NLR, PLR, SII, MHR, and CAR values were observed in patients with orbitopathy and showed significant correlations with TRAb, TSI, Clinical Activity Score, and Hertel measurements. Among these markers, MHR and CAR demonstrated particularly strong predictive performance, while a combined inflammatory model incorporating NLR, SII, MHR, and CAR achieved excellent discrimination for identifying Graves’ orbitopathy. These findings suggest that readily available inflammatory indices may serve as useful complementary biomarkers for risk assessment and clinical evaluation in patients with Graves’ disease. Further prospective multicenter studies are warranted to validate these findings and establish their role in routine clinical practice.

## Figures and Tables

**Table 1:** Baseline demographic, clinical, and thyroid-related characteristics of the study population (*n* = 178).

Variable	Overall Cohort (*n* = 178)
Age (years)	41.35 ± 12.06
Female gender, *n* (%)	146 (82.0%)
Male gender, *n* (%)	32 (18.0%)
Current smoker, *n* (%)	33 (18.5%)
Graves’ disease and treatment characteristics	
TRAb-positive Graves’ disease, *n* (%)	154 (86.5%)
Newly diagnosed patients, *n* (%)	8 (4.5%)
Antithyroid drug therapy, *n* (%)	167 (93.8%)
Methimazole therapy, *n* (%)	160 (89.9%)
Propylthiouracil therapy, *n* (%)	7 (3.9%)
Radioactive iodine therapy, *n* (%)	6 (3.4%)
Thyroidectomy, *n* (%)	5 (2.8%)
Graves’ orbitopathy, *n* (%)	63 (35.4%)
Orbitopathy activity and severity parameters	
Clinical Activity Score (CAS) *	2.35 ± 1.71
Right Hertel measurement (mm) *	21.02 ± 2.18
Left Hertel measurement (mm) *	21.08 ± 2.09

Data are presented as mean ± standard deviation, median (interquartile range), or number (percentage), as appropriate. * CAS and Hertel measurements were available for patients who underwent ophthalmological evaluation. CAS, Clinical Activity Score; TRAb, thyrotropin receptor antibody.

**Table 2:** Baseline thyroid-related, hematological, and inflammatory characteristics of the study population (*n* = 178).

Variable	Overall Cohort (*n* = 178)
Thyroid imaging and antibody parameters	
Thyroid volume (mL)	11.38 (7.60–17.61)
Isthmus thickness (mm)	4.86 ± 1.73
TRAb at admission (IU/L)	4.32 (2.33–9.87)
TSI	4.00 (1.93–10.50)
Anti-TPO (IU/mL)	226.05 (38.47–681.12)
Anti-Tg (IU/mL)	6.05 (0.93–40.98)
Baseline thyroid function tests	
TSH (mIU/L)	0.28 (0.01–1.84)
FT3 (pg/mL)	3.71 (3.25–4.94)
FT4 (ng/dL)	0.82 (0.67–1.42)
FT3/FT4 ratio	3.99 (3.04–5.01)
Baseline lipid and hematological parameters	
HDL cholesterol (mg/dL)	52.55 ± 13.83
Neutrophil count (×10^9^/L)	4.17 (3.42–5.39)
Lymphocyte count (×10^9^/L)	2.59 ± 0.87
Platelet count (×10^9^/L)	295.35 ± 83.52
Monocyte count (×10^9^/L)	0.59 (0.48–0.70)
Baseline inflammatory and biochemical indices	
Albumin (g/L)	42.54 ± 4.14
CRP (mg/L)	2.50 (1.30–4.47)
NLR	1.78 (1.33–2.33)
PLR	121.80 ± 43.98
SII	495.94 (360.44–707.85)
MHR	0.66 ± 0.60
CAR	0.06 (0.04–0.10)

Data are presented as mean ± standard deviation, median (interquartile range), or number (percentage), as appropriate. TRAb, thyrotropin receptor antibody; TSI, thyroid-stimulating immunoglobulin; Anti-TPO, anti-thyroid peroxidase antibody; Anti-Tg, anti-thyroglobulin antibody; TSH, thyroid-stimulating hormone; FT3, free triiodothyronine; FT4, free thyroxine; HDL, high-density lipoprotein cholesterol; CRP, C-reactive protein; NLR, neutrophil-to-lymphocyte ratio; PLR, platelet-to-lymphocyte ratio; SII, systemic immune-inflammation index; MHR, monocyte-to-HDL cholesterol ratio; CAR, C-reactive protein-to-albumin ratio.

**Table 3:** Comparison of demographic, thyroid-related, ophthalmological, and inflammatory characteristics according to Graves’ orbitopathy status.

Variable	Orbitopathy (+) (*n* = 63)	Orbitopathy (−) (*n* = 115)	*p*-Value
Age (years)	43.8 ± 11.7	40.0 ± 12.1	0.038
Female gender, *n* (%)	54 (85.7)	92 (80.0)	0.344
Current smoker, *n* (%)	18 (28.6)	15 (13.0)	0.012
Disease duration (months)	28 (12–54)	16 (8–36)	0.011
TRAb (IU/L)	8.92 (4.65–16.33)	3.14 (1.86–6.72)	<0.001
TSI	9.40 (4.10–18.60)	2.90 (1.40–7.50)	<0.001
Anti-TPO (IU/mL)	284 (72–715)	201 (35–612)	0.128
Anti-Tg (IU/mL)	8.4 (1.4–55.2)	5.1 (0.8–34.5)	0.203
TSH (mIU/L)	0.03 (0.01–0.52)	0.09 (0.01–2.11)	0.084
FT3 (pg/mL)	4.64 (3.54–7.10)	3.42 (3.12–4.31)	0.002
FT4 (ng/dL)	1.18 (0.82–2.31)	0.75 (0.65–1.18)	0.004
FT3/FT4 ratio	4.46 ± 2.81	4.12 ± 2.27	0.421
Ophthalmological characteristics			
Clinical Activity Score (CAS)	3.82 ± 1.34	0.00 ± 0.00	<0.001
Clinical severity score	2.48 ± 0.92	0.00 ± 0.00	<0.001
Right Hertel measurement (mm)	22.7 ± 2.3	20.1 ± 1.4	<0.001
Left Hertel measurement (mm)	22.8 ± 2.2	20.2 ± 1.3	<0.001
Diplopia, *n* (%)	20 (31.7)	0 (0.0)	<0.001
Ocular motility restriction, *n* (%)	20 (31.7)	0 (0.0)	<0.001
Hematological parameters			
Neutrophil count (×10^9^/L)	4.91 (3.92–6.23)	3.88 (3.21–4.87)	<0.001
Lymphocyte count (×10^9^/L)	2.31 ± 0.79	2.74 ± 0.88	0.003
Platelet count (×10^9^/L)	309 ± 86	288 ± 80	0.114
Monocyte count (×10^9^/L)	0.66 (0.53–0.78)	0.55 (0.45–0.67)	0.001
HDL cholesterol (mg/dL)	49.1 ± 12.5	54.4 ± 14.2	0.019
Inflammatory markers			
NLR	2.18 (1.71–2.98)	1.53 (1.20–1.97)	<0.001
PLR	139.6 ± 46.5	112.8 ± 39.2	<0.001
SII	692.4 (498.2–955.6)	418.6 (311.5–578.2)	<0.001
MHR	0.89 ± 0.48	0.54 ± 0.32	<0.001
CAR	0.10 (0.06–0.17)	0.05 (0.03–0.08)	<0.001

Data are presented as mean ± standard deviation, median (interquartile range), or number (%), as appropriate. TRAb, thyrotropin receptor antibody; TSI, thyroid-stimulating immunoglobulin; Anti-TPO, anti-thyroid peroxidase antibody; Anti-Tg, anti-thyroglobulin antibody; TSH, thyroid-stimulating hormone; FT3, free triiodothyronine; FT4, free thyroxine; CAS, Clinical Activity Score; HDL, high-density lipoprotein cholesterol; NLR, neutrophil-to-lymphocyte ratio; PLR, platelet-to-lymphocyte ratio; SII, systemic immune-inflammation index; MHR, monocyte-to-HDL cholesterol ratio; CAR, C-reactive protein-to-albumin ratio.

**Table 4:** Correlations between inflammatory markers and thyroid autoimmunity parameters.

Parameter	NLR (r)	*p*-Value	PLR (r)	*p*-Value	SII (r)	*p*-Value	MHR (r)	*p*-Value	CAR (r)	*p*-Value
TRAb	0.382	<0.001	0.294	<0.001	0.431	<0.001	0.417	<0.001	0.351	<0.001
TSI	0.346	<0.001	0.271	0.001	0.402	<0.001	0.388	<0.001	0.322	<0.001
TSH	−0.219	0.004	−0.171	0.023	−0.248	0.001	−0.204	0.007	−0.183	0.015
FT3	0.287	<0.001	0.216	0.004	0.335	<0.001	0.298	<0.001	0.243	0.001
FT4	0.254	0.001	0.188	0.012	0.301	<0.001	0.272	<0.001	0.211	0.005
FT3/FT4 ratio	0.091	0.228	0.067	0.377	0.102	0.177	0.084	0.266	0.073	0.335
Anti-TPO	0.118	0.118	0.097	0.198	0.126	0.094	0.134	0.075	0.082	0.279
Anti-Tg	0.086	0.254	0.052	0.492	0.093	0.218	0.075	0.321	0.061	0.421
CAS	0.446	<0.001	0.341	<0.001	0.523	<0.001	0.478	<0.001	0.412	<0.001
Right Hertel	0.318	<0.001	0.236	0.002	0.387	<0.001	0.352	<0.001	0.298	<0.001
Left Hertel	0.326	<0.001	0.241	0.001	0.394	<0.001	0.361	<0.001	0.305	<0.001

Spearman rank correlation analysis was used. TRAb, thyrotropin receptor antibody; TSI, thyroid-stimulating immunoglobulin; TSH, thyroid-stimulating hormone; FT3, free triiodothyronine; FT4, free thyroxine; Anti-TPO, anti-thyroid peroxidase antibody; Anti-Tg, anti-thyroglobulin antibody; CAS, Clinical Activity Score; NLR, neutrophil-to-lymphocyte ratio; PLR, platelet-to-lymphocyte ratio; SII, systemic immune-inflammation index; MHR, monocyte-to-HDL cholesterol ratio; CAR, C-reactive protein-to-albumin ratio.

**Table 5:** Univariable and multivariable logistic regression analyses for predictors of Graves’ orbitopathy.

Variable	Univariable OR (95% CI)	*p*-Value	Multivariable OR (95% CI)	*p*-Value
Age	1.03 (1.00–1.06)	0.041	1.02 (0.99–1.05)	0.118
Female gender	1.50 (0.64–3.51)	0.346	—	—
Current smoker	2.66 (1.22–5.82)	0.014	2.31 (1.01–5.28)	0.047
TRAb	1.12 (1.06–1.19)	<0.001	1.08 (1.02–1.15)	0.009
TSI	1.10 (1.05–1.16)	<0.001	1.06 (1.00–1.12)	0.041
FT3	1.31 (1.10–1.56)	0.003	0.95 (0.74–1.22)	0.709
FT4	1.42 (1.12–1.81)	0.004	1.60 (0.74–3.45)	0.231
NLR	2.18 (1.48–3.21)	<0.001	1.63 (1.04–2.56)	0.034
PLR	1.02 (1.01–1.03)	<0.001	0.99 (0.97–1.00)	0.155
SII	1.01 (1.00–1.01)	<0.001	1.01 (1.00–1.01)	0.018
MHR	4.82 (2.34–9.93)	<0.001	2.91 (1.26–6.72)	0.012
CAR	6.75 (2.81–16.23)	<0.001	3.84 (1.42–10.39)	0.008

Data are presented as odds ratios (ORs) with 95% confidence intervals (CIs). Variables with *p* < 0.10 in univariable analysis were considered for multivariable logistic regression. TRAb, thyrotropin receptor antibody; TSI, thyroid-stimulating immunoglobulin; FT3, free triiodothyronine; FT4, free thyroxine; NLR, neutrophil-to-lymphocyte ratio; PLR, platelet-to-lymphocyte ratio; SII, systemic immune-inflammation index; MHR, monocyte-to-HDL cholesterol ratio; CAR, C-reactive protein-to-albumin ratio.

**Table 6:** ROC analysis of inflammatory markers for predicting Graves’ orbitopathy.

Marker	AUC	95% CI	Cut-Off	Sensitivity (%)	Specificity (%)	Youden Index	*p*-Value
NLR	0.741	0.666–0.815	1.89	71.4	68.7	0.401	<0.001
PLR	0.703	0.624–0.782	126.5	68.3	63.5	0.318	<0.001
SII	0.792	0.724–0.860	562.4	76.2	72.2	0.484	<0.001
MHR	0.818	0.754–0.882	0.74	79.4	73.0	0.524	<0.001
CAR	0.774	0.702–0.846	0.08	73.0	71.3	0.443	<0.001
Combined model *	0.891	0.842–0.940	≥0.43 **	84.1	82.6	0.667	<0.001
DeLong comparison of AUCs							
Comparison			ΔAUC		*p*-value		
Combined model vs. NLR			0.150		<0.001		
Combined model vs. PLR			0.188		<0.001		
Combined model vs. SII			0.099		0.011		
Combined model vs. MHR			0.073		0.047		
Combined model vs. CAR			0.117		0.008		

* The combined inflammatory model included NLR, PLR, SII, MHR, and CAR. ** Predicted probability cut-off. AUC, area under the curve; CI, confidence interval; NLR, neutrophil-to-lymphocyte ratio; PLR, platelet-to-lymphocyte ratio; SII, systemic immune-inflammation index; MHR, monocyte-to-HDL cholesterol ratio; CAR, C-reactive protein-to-albumin ratio.

**Table 7:** Distribution of NLR, PLR, SII, MHR, and CAR according to CAS categories.

CAS Category	*n*	NLR	PLR	SII	MHR	CAR
CAS 0–1	115	1.53 (1.20–1.97)	112.8 ± 39.2	418.6 (311.5–578.2)	0.54 ± 0.32	0.05 (0.03–0.08)
CAS 2–3	25	1.89 (1.45–2.41)	126.4 ± 41.7	560.2 (430–710)	0.69 ± 0.37	0.07 (0.04–0.11)
CAS 4–5	23	2.42 (1.90–3.15)	148.7 ± 44.5	760.5 (590–980)	0.96 ± 0.49	0.11 (0.07–0.18)
CAS ≥ 6	15	2.95 (2.20–3.80)	167.3 ± 48.2	980.8 (760–1250)	1.18 ± 0.55	0.15 (0.10–0.24)
*p*-value	—	<0.001	<0.001	<0.001	<0.001	<0.001

## Data Availability

The original contributions presented in this study are included in the article. Further inquiries can be directed to the corresponding author.

## Abbreviations

Anti-Tg	Anti-thyroglobulin antibody
Anti-TPO	Anti-thyroid peroxidase antibody
AUC	Area under the curve
CAR	C-reactive protein-to-albumin ratio
CAS	Clinical Activity Score
CI	Confidence interval
CRP	C-reactive protein
EUGOGO	European Group on Graves’ Orbitopathy
FT3	Free triiodothyronine
FT4	Free thyroxine
GD	Graves’ disease
GO	Graves’ orbitopathy
HDL	High-density lipoprotein
MHR	Monocyte-to-high-density lipoprotein cholesterol ratio
NLR	Neutrophil-to-lymphocyte ratio
OR	Odds ratio
PLR	Platelet-to-lymphocyte ratio
ROC	Receiver operating characteristic
SII	Systemic immune-inflammation index
TSH	Thyroid-stimulating hormone
TRAb	Thyrotropin receptor antibody
TSHR	Thyroid-stimulating hormone receptor
TSI	Thyroid-stimulating immunoglobulin
VIF	Variance inflation factor
